# A Comparison of Commercially Available Screen-Printed Electrodes for Electrogenerated Chemiluminescence Applications

**DOI:** 10.3389/fchem.2020.628483

**Published:** 2021-01-28

**Authors:** Emily Kerr, Richard Alexander, Paul S. Francis, Rosanne M. Guijt, Gregory J. Barbante, Egan H. Doeven

**Affiliations:** ^1^ Institute for Frontier Materials, Deakin University, Geelong, VIC, Australia; ^2^ Centre for Regional and Rural Futures, Deakin University, Geelong, VIC, Australia; ^3^ School of Life and Environmental Sciences, Deakin University, Geelong, VIC, Australia; ^4^ ARC Training Centre for Portable Analytical Separation Technologies (ASTech), Future Industries Institute, University of South Australia, Mawson Lakes, SA, Australia

**Keywords:** electrogenerated, chemiluminescence, screen-printed electrodes, point-of-care sensors, electrochemiluminescence

## Abstract

We examined a series of commercially available screen-printed electrodes (SPEs) for their suitability for electrochemical and electrogenerated chemiluminescence (ECL) detection systems. Using cyclic voltammetry with both a homogeneous solution-based and a heterogeneous bead-based ECL assay format, the most intense ECL signals were observed from unmodified carbon-based SPEs. Three commercially available varieties were tested, with Zensor outperforming DropSens and Kanichi in terms of sensitivity. The incorporation of nanomaterials in the electrode did not significantly enhance the ECL intensity under the conditions used in this evaluation (such as gold nanoparticles 19%, carbon nanotubes 45%, carbon nanofibers 21%, graphene 48%, and ordered mesoporous carbon 21% compared to the ECL intensity of unmodified Zensor carbon electrode). Platinum and gold SPEs exhibited poor relative ECL intensities (16% and 10%) when compared to carbonaceous materials, due to their high rates of surface oxide formation and inefficient oxidation of tri-*n*-propylamine (TPrA). However, the ECL signal at platinum electrodes can be increased ∼3-fold with the addition of a surfactant, which enhanced TPrA oxidation due to increasing the hydrophobicity of the electrode surface. Our results also demonstrate that each SPE should only be used once, as we observed a significant change in ECL intensity over repeated CV scans and SPEs cannot be mechanically polished to refresh the electrode surface.

## Introduction

The personal glucometer is a device that employs screen-printed electrodes (SPEs) to provide diabetic patients with accurate blood glucose measurements using an electrochemical reaction. It is one of many examples of electrochemical point-of-care (POC) diagnostic systems, with other analytical targets including heavy metals, pesticides, ethanol, dopamine, nucleic acids or specific antigens (Boujtita et al., 2000; Darain et al., 2003; Carpini et al., 2004; Kadara and Tothill, 2004; Arduini et al., 2006; Martinez et al., 2009; Viswanathan et al., 2009; Nie et al., 2010a; Nie et al., 2010b; Metters et al., 2011; Ping et al., 2012; Chen et al., 2019). Electrogenerated chemiluminescence also referred to as electrochemiluminescence (ECL) provides a highly sensitive mode of detection (Miao et al., 2002; Miao and Bard, 2003, Miao and Bard, 2004; Miao, 2008; Chen et al., 2019), measuring the light emission at an electrode surface arising from the formation of excited states as a result of an electrochemical reaction (Richter, 2004; Richter, 2008). ECL offers many advantages when compared to conventional electrochemical, fluorescent or chemiluminescent detection techniques as it does not require precise current monitoring or an external light source; ECL also offers accurate spatial and temporal control over the reaction. Following their success in electrochemical assays, ECL detection strategies have been developed using disposable SPEs, aiming to combine the enhanced sensitivity with simplified and low-cost devices, addressing drawbacks of many proof-of-concept POC devices (Delaney et al., 2011; Ge et al., 2012; Xu and Wang, 2012; Yan et al., 2012; Delaney et al., 2013; Doeven et al., 2015a; Chen et al., 2019). A recent review by Martínez-Periñán et al. (2020) evaluated the plethora of analytical applications of ECL sensing using SPEs.

Numerous electrode characteristics are important for developing highly sensitive analytical ECL and electrochemical diagnostic applications, including: 1) fast electron transfer rates; 2) highly reproducible electrode surfaces to improve assay precision; 3) high electroactive surface area to maximize signal; 4) high electrode surface stability, to improve the reproducibility between potential scans and prevent electrode passivation; 5) wide electrochemical potential window; 6) low background current; and 7) for ECL, a hydrophobic electrode surface, to permit efficient oxidation of the co-reactant TPrA (Workman and Richter, 2000; Li and Zu, 2004; Valenti et al., 2016). Valenti et al. (2016) recently reviewed classical electrode materials and defined these critical parameters that electro-active materials must possess to produce efficient ECL with the classic tris(2,2′-bipyridine)ruthenium(II) ([Ru(bpy)_3_]^2+^) and tri-*n*-propylamine (TPrA) oxidative-reduction co-reactant system. Kadara et al. (2009) and Fanjul-Bolado et al. (2008) conducted thorough electrochemical characterizations of a range of in-house produced and commercially available SPEs. Since then, a wide variety of SPEs that exploit advances in electro-active materials, such as modified electrodes incorporating carbon nanofibers (CNF), carbon nanotubes (CNT), gold nanoparticles (GNP), graphene (GPH), ordered mesoporous carbon (OMC) and combinations of these materials, have become available from commercial suppliers (Zhao et al., 2002; Kim and Yang, 2003; Chang et al., 2006; Guo and Wang, 2007; Huang et al., 2008; Liu et al., 2008; Brownson and Banks, 2011). Herein, we interrogate 13 commercially available SPEs including traditional (carbon, gold and platinum) and modified electrodes for their suitability for use for ECL detection in a POC setting.

## Experimental

### Chemicals

Potassium hexacyanoferrate(II) (potassium ferrocyanide, >98%, Ajax, Australia), potassium chloride (>99%, LabServ, Australia), sodium chloride (>99.5%, Sigma Aldrich), potassium phosphate monobasic (>99%, Sigma-Aldrich), potassium phosphate dibasic (>98%, Sigma Aldrich), Tris (>99%, Sigma-Aldrich), borate (>99%, Sigma-Aldrich) potassium hydroxide (>85%, Sigma-Aldrich), hydrochloric acid (32%, Chem-Supply, Australia), tri-*n*-propylamine (>98%, Sigma-Aldrich), tris(2,2′-bipyridine)ruthenium(II) chloride hexahydrate ([Ru(bpy)_3_]Cl_2_.6H_2_O, >99.5%, Sigma-Aldrich), Tween 20 (polyethylene glycol sorbitan monolaurate, T20, Sigma-Aldrich), and Triton X-100 (polyethylene glycol tert octylphenyl ether, TX-100, Ajax) were used as supplied. All samples were prepared in milli Q water (18.2 MΩ cm^−1^). Biotinylated 89mer ssDNA used as a nucleic acid proxy assay was purchased from Integrated DNA Technologies, United States (details in SI), Bis(2,2′-bipyridine)(4-methyl-4′-carboxypropyl-2,2′-bipyridine)ruthenium(II) hexafluorophosphate (Ru(bpy)_2_([mbpy-COOH](PF_6_)_2_) was purchased from SunaTech. Dynabeads d280 streptavidin coated 2.8 µm paramagnetic beads (Invitrogen) were purchased from Life Technologies (Australia), and were washed in binding buffer (0.5 M NaCl, 20 mM Tris.HCl, pH 8.0) three times prior to use. Beads were used at a concentration of 2 mg/mL and stored in binding buffer unless otherwise specified.

### Analysis Procedures

We machined custom SPE holders from 10 mm thick cast poly(methyl methacrylate) (PMMA) sheets using a Datron M7HP CNC mill (Datron AG, Germany). SPE holders were designed using SolidWorks 2015 CAD package (Dassault Systems, France), while G-code CNC toolpaths were created using Siemens NX 10 CAD/CAM package (Siemens, Germany). 3D drawings of these holders are shown in Supplementary Figure S1. These holders were designed to house the SPEs for analysis and to reproducibly interface the cells with the detector. For the paramagnetic particle based experiments, the holders were designed to hold a 3 × 4 mm diameter rod shaped N42 rare earth magnet (Aussie Magnets, Australia) beneath the working electrode position, to facilitate particle capture at the electrode surface. Two detectors were used: solution phase ECL was detected using a photomultiplier tube (extended-range trialkali S20 PMT, ET Enterprises model 9828B), while ECL from the magnetic bead based assay was detected using an AdvanSiD (Italy) 3 × 3 mm silicon photon multiplier (ASD-RGB3S-P), to remove any effect of the magnetic field on the PMT. The SiPM was biased at 33 V and interfaced with an AdvanSiD ASD EP EB N amplifier board. Data from the SiPM was recorded using an eDAQ401 (eDAQ, Australia) data recording unit using the supplied eDAQ Chart software.

For electrochemical and ECL (PMT) experiments, we used a custom-built, light-tight, Faraday cage and an Autolab PGSTAT 101 or PGSTAT 128 N (Metrohm Autolab B.V., Netherlands) potentiostat with accompanying NOVA software. The following varieties of electrodes were obtained from DropSens (http://www.dropsens.com/): unmodified carbon (DS-C), ordered mesoporous carbon (DS-OMC), carbon nanotubes (DS-CNT), carbon nanofiber (DS-CNF), graphene (DS-GPH), gold (BT-250 model, DS-Au) platinum (DS-Pt), carbon with gold nanoparticles (DS-GNP), carbon nanofiber with gold nanoparticles (DS-CNF-GNP), carbon nanotubes with gold nanoparticles (DS-CNT-GNP), and graphene with gold nanoparticles (DS-GPH-GNP). Two additional varieties of carbon electrodes were obtained from eDAQ (http://www.edaq.com/, Zensor and Kanichi varieties). Each SPE contained a three-electrode configuration with varying working electrode surfaces (DropSens varieties 4 mm working electrode diameter, Kanichi and Zensor, 3 mm working electrode diameter), carbon auxiliary electrode and Ag/AgCl reference electrode. For electrochemical experiments, analytes were measured in either 1 M KCl or 0.1 M phosphate buffer solution (PBS), pH 7.5. For ECL experiments, solutions of analyte at the appropriate concentration were prepared in ECL buffer; 0.1 M PBS, pH 7.5, containing 100 mM TPrA. Relative ECL intensities were calculated from the integrated area of the PMT response from three cyclic voltammetry (CV) cycles between 1.6 V and −1.2 V at 0.1 V/s and each result was proportionally adjusted to the geometric working electrode surface area.

For DNA assay comparison experiments, we prepared an Ru-DNA-biotin construct that could be immobilized on paramagnetic particles, following the procedure detailed by Zhou et al. (2014). Ru(bpy)_2_(mbpy-NHS)^2+^ was prepared from Ru(bpy)_2_(mbpy-COOH)^2+^ as previously described, followed by bioconjugation with the NH_2_ terminated DNA sequence (Zhou et al., 2014; Chen et al., 2019). The DNA was isolated and the Ru-DNA-biotin conjugate concentration quantified by UV-visible spectrometry. This purified Ru-DNA-biotin conjugate was then bound to the paramagnetic beads by streptavidin-biotin interaction. The beads (with bound Ru-DNA-biotin construct) were then washed and re-suspended in binding buffer at 2 mg/mL for later use.

To perform ECL experiments on the Ru-DNA-bead constructs, the paramagnetic beads were re-suspended in ECL buffer at 2 mg/mL. The SPE to be tested was mounted in the holder, then 80 µL of ECL buffer was pipetted into the well in the holder located over the electrode area. 5 µL of the Ru-DNA-bead solution (10 µG paramagnetic particles) was then pipetted over the working electrode area, where the beads were captured at the working electrode surface by the magnet located directly underneath. The detector was then mounted to the top of the cell (the SiPM fits in the machined recess) and ECL was performed in an identical fashion to the solution phase experiments.

We employed a handheld digital multi-meter to measure the conductive path resistance (Dick Smith Electronics, Q-1559). The measurement was taken from the working electrode connection pad to the center of the working electrode surface. An acceleration voltage of 12 kV and either an in-lens or secondary electron (SE) detector was employed to collect scanning electron micrographs (Zeiss Supra 55VP Scanning Electron Microscope, SEM; Zeiss, Germany). The working distance (WD) used to collect the SEM micrographs was optimized for each electrode and was between 3.2 and 4.7 mm. Contact angles for each electrode were collected using a contact angle goniometer (Ramè-Hart, United States).

## Results and Discussion

### Electrochemical Properties

We tested each electrode for its electrochemical properties (Table 1) using potassium ferro/ferricyanide, a thoroughly studied outer-sphere redox couple. The peak-to-peak separation of the redox couple (Δ*E*) for each electrode was greater than anticipated for a one-electron transfer process (59 mV), displaying quasi-reversible electrochemical characteristics. Although not ideal, these results are consistent with those of both Fanjul-Bolado et al. (2008) and Banks et al. (2005) who proposed that the electrochemical irreversibility of the ferro/ferricyanide couple at these electrode surfaces results from a combination of electrode characteristics including the nature of the ink used to produce the electrode, the amount of organic binder incorporated into the electrode, the temperature employed in the curing process, the degree of formation of oxygenated species at the electrode surface, the hydrophilicity of the electrode surface and the electrode material itself (Fanjul-Bolado et al., 2008; Kadara et al., 2009).

**TABLE 1:** Electrochemical properties of various commercially available SPEs.

	*A* (cm^2^)^a^ ^,^ ^b^	*A*_*real*_ ^c^	Conductive path resistance (Ω)^d^	Δ*E* (mV)	I_c_/I_a_	TPrA oxidation (µmol/cm^2^)^e^
Zensor	0.027	0.38	115	131	1.05	1.11
DS-C	0.055	0.44	477	156	1.00	1.20
Kanichi	0.040	0.56	196	126	1.01	0.98
DS-OMC	0.056	0.44	262	76	0.96	0.65
DS-CNT	0.078	0.62	279	81	0.99	1.22
DS-CNF	0.098	0.78	376	81	1.01	1.16
DS-GPH	0.073	0.58	353	91	0.97	0.85
DS-Pt	0.099	0.79	1	65	1.01	0.24
DS-Au	0.083	0.66	1	70	0.99	3.38
DS-GNP	0.069	0.55	254	111	1.06	1.08
DS-CNF-GNP	0.103	0.82	202	70	0.99	1.22
DS-GPH-GNP	0.114	0.91	259	70	1.00	1.39
DS-CNT-GNP	0.080	0.64	291	91	1.05	1.12
GC	0.051	0.72	4	70	1.02	3.37

^a^ Calculated using Eq. 1, (average RSD = 5%, n = 3).

^b^ Also commonly referred to as ‘roughness factor’.

^c^ Calculated using Eq. 2.

^d^ Average RSD = 2%, n = 3.

^e^ Calculated using N=(Q/nFA), where *Q* is the charge at the electrode surface (the area under the blank subtracted TPrA anodic oxidation wave current vs time peak, in coulombs), n is the number of electrons transferred in the reaction, F is the Faraday constant (96,485 C mol^−1^) and A is the geometric electrode area (cm^2^), (average RSD = 7%, n = 2).

The electro-active area (*A*) of the electrode was calculated from scan rate studies of 1 mM potassium ferrocyanide in 1 M KCl, using the Randles-Sevcik Eq. 1 (Bard and Faulkner, 1980).

$$i_{p}=\left(2.69\times 10^{5}\right)n^{3/2}ACD^{1/2}v^{1/2},$$ (1)

where *i*_*p*_ is the peak current, *n* is the number of electrons participating in the electron transfer reaction, *A* is the working electrode area, *D* is the diffusion coefficient and *v* is the scan rate. We have included representative scan rate studies and graphs of the variation of *i*_*p*_ with scan rate in Supplementary Figures S2, S3. The proportion of the electrode surface that is electro-active (*A*_real_) can be calculated using the following equation;

$$A_{real}=\frac{A}{A_{geo}},$$ (2)

where *A*_geo_ is the geometric area of the electrode. As expected, the nanomaterial modified electrodes generally showed an increase in *A*_real_ when compared to standard carbon electrodes (with the exception of DS-OMC), and electrodes modified with two different nano-materials (e.g., nanotubes and gold nano-particles) exhibited a further increase in *A*_real_. High resistance can adversely affect the reversibility of electron transfer reactions at the electrode surface (Keil, 1986). We observed minimal resistance in both DS-Au and DS-Pt electrodes, due to the high conductivity of gold and platinum metals. Carbon electrodes showed much higher resistance. The Zensor variety displayed the lowest resistivity (115 Ω) of the carbon electrodes. The resistivity of the DropSens carbon-based varieties varied between 202 and 477 Ω. The Kanichi electrodes, unlike DropSens and Zensor varieties, do not have an underlying silver track between the working electrodes and the connectors and displayed the highest resistivity at 1966 Ω.

### SEM Characterization

**FIGURE 1:**
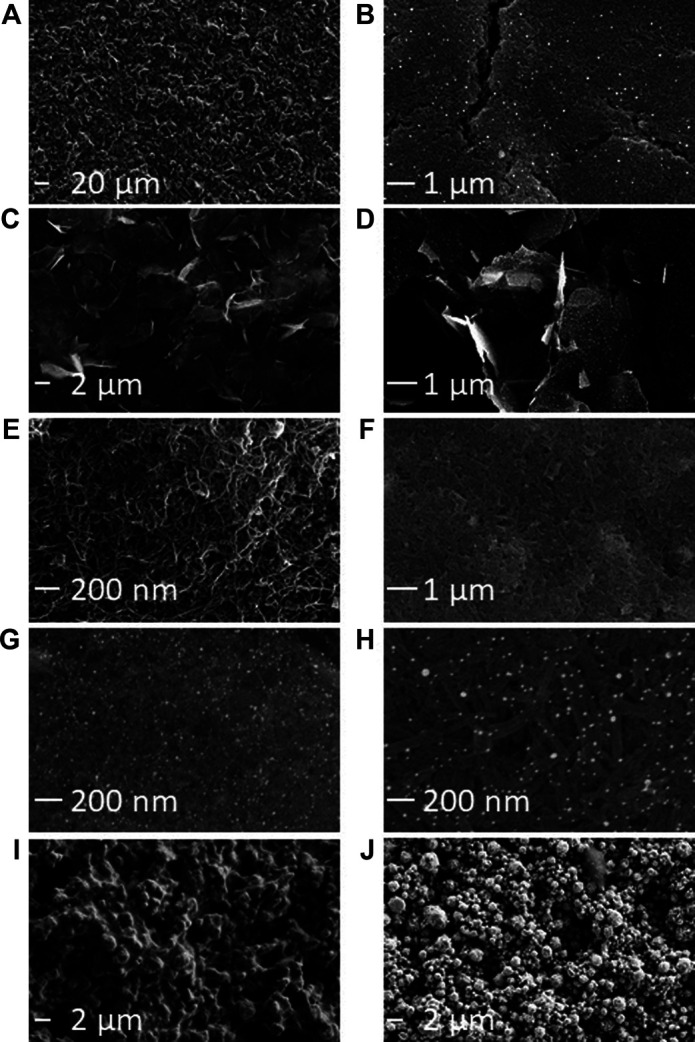
SEM characterization of commercially available SPE varieties. **(A)** Kanichi. **(B)** DS-GNP. **(C)** DS-GPH. **(D)** DS-GPH-GNP. **(E)** DS-CNT. **(F)** DS-CNF. **(G)** DS-CNT-GNP. **(H)** DS-CNF-GNP. **(I)** DS-Pt. **(J)** DS-Au. Additional SEM micrographs are included in Supplementary Figure S2. An SE detector was used to collect micrographs A, I, and J. An in-lens detector was used for all other micrographs.

It is possible to visualize the electroactive surface area on each electrode variety using the SEM micrographs, as the exposed edge or plane-like surfaces are the predominant source of electron transfer in carbon-based electrodes (Banks et al., 2005; Kadara et al., 2009). SEM micrographs showed variations between the different modified electrodes (Figure 1; Supplementary Figure S4). Unmodified Kanichi, DropSens and Zensor carbon electrodes displayed a similar surface profile, with visible graphitic particles surrounded by a binding polymer, as previously observed by Kadara et al. (2009). Carbon electrodes modified with GNPs showed a similar surface structure to DS-C electrodes with the addition of GNPs present on the surface ranging between ∼30–100 nm in diameter. Graphene modified electrodes exhibited distinct graphene ‘shards’ and electrodes modified with both graphene and GNPs displayed spherical GNPs distributed across the graphene shards. Electrodes modified with carbon nanotubes and carbon nanofibers both exhibited ‘web-like’ appearances and the respective GNP derivatives showed spherical GNPs embedded in the web-like surface. Platinum and gold electrodes both displayed distinct metallic crystalline ‘bead’ structures.

### ECL

**FIGURE 2:**
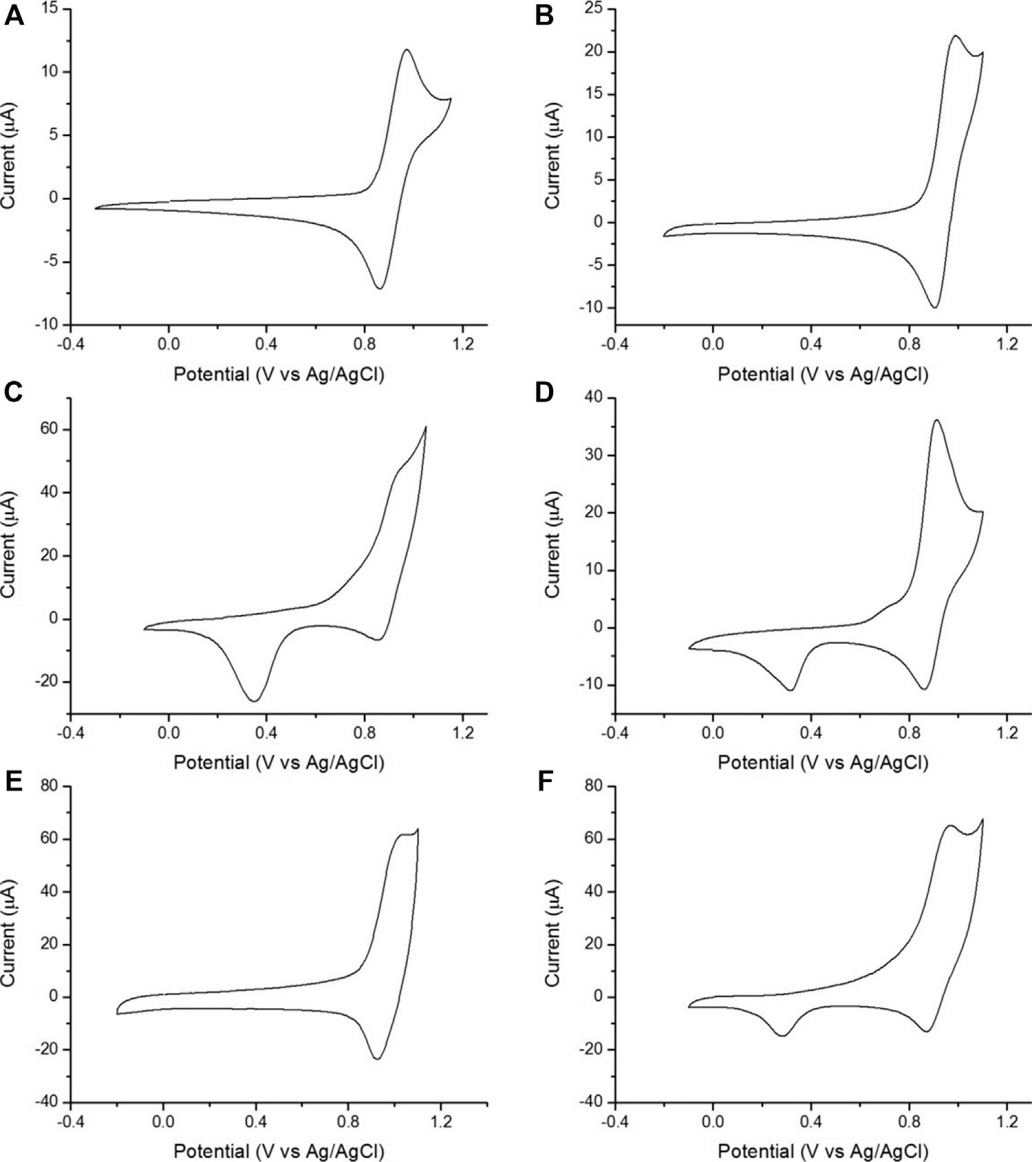
Representative CVs of 1 mM [Ru(bpy)_3_]^2+^ in 0.1 M PBS (scan rate 0.1 V/s). **(A)** Kanichi. **(B)** DS-CNT. **(C)** DS-Au. **(D)** DS-GNP. **(E)** DS-GPH. **(F)** DS-GPH-GNP.

CVs of [Ru(bpy)_3_]^2+^ in PBS showed a reversible oxidation peak at 0.92 V vs Ag/AgCl (representative CVs are shown in Figure 2. For all CVs, see Supplementary Figure S5). CVs of gold or gold-nanoparticle-modified electrodes also exhibited additional waves corresponding to the formation of gold oxides at the electrode surface. The formation of surface oxides began at ∼0.7 V vs Ag/AgCl (appearing as a shoulder on the [Ru(bpy)_3_]^2+^ oxidation wave) and in the reverse sweep, we observed the corresponding reduction of the surface oxide layer at ∼0.3 V vs Ag/AgCl (Woods and Bard, 1976; Bard and Faulkner, 1980; Zu and Bard, 2000). We also collected CVs of TPrA at 100 mM (Supplementary Figure S6) to monitor the extent of TPrA oxidation, an important, rate-limiting step in ECL (Miao et al., 2002; Choi and Bard, 2005).

To compare the selected SPEs to determine their relative ECL intensities, we selected relatively low [Ru(bpy)_3_]^2+^ concentrations (1 × 10^−7^ M and 1 × 10^−8^ M) to reflect the low concentrations of metal complex present in bioanalytical ECL applications (Santhanam and Bard, 1965; Zu and Bard, 2000; Miao et al., 2002). The generalized mechanism for [Ru(bpy)_3_]^2+^ ECL with TPrA is outlined in reaction steps 1–9 below:

1. [Ru(bpy)_3_]^2+^–e^−^→[Ru(bpy)_3_]^3+^
2. TPrA–e^−^→TPrA^+•^
3. [Ru(bpy)_3_]^3+^ + TPrA→[Ru(bpy)_3_]^2+^ + TPrA^+•^
4. TPrA^•+^→TPrA^•^ + H^+^
5. [Ru(bpy)_3_]^3+^ + TPrA^•^ → [Ru(bpy)_3_]^2+^* + Pr_2_N^+^C=H_2_CH_3_
6. [Ru(bpy)_3_]^2+^ + TPrA^•^ → [Ru(bpy)_3_]^+^ + Pr_2_N^+^C=H_2_CH_3_
7. [Ru(bpy)_3_]^3+^ + [Ru(bpy)_3_]^+^→[Ru(bpy)_3_]^2+^* + [Ru(bpy)_3_]^2+^
8. [Ru(bpy)_3_]^+^ + TPrA^•+^→[Ru(bpy)_3_]^2+^* + TPrA
9. [Ru(bpy)_3_]^2+^*→[Ru(bpy)_3_]^2+^ + *hv* (*λ*_max_ = 620 nm)

In magnetic bead based assays, where [Ru(bpy)_3_]^2+^ is located too far away from the electrode to undergo direct oxidation, and at low concentrations of [Ru(bpy)_3_]^2+^, the amount of formation of [Ru(bpy)_3_]^3+^ via reaction 1 is small and ECL is predominantly produced by reactions 2, 4, 6, 8, and 9 (Miao et al., 2002).

**FIGURE 3:**
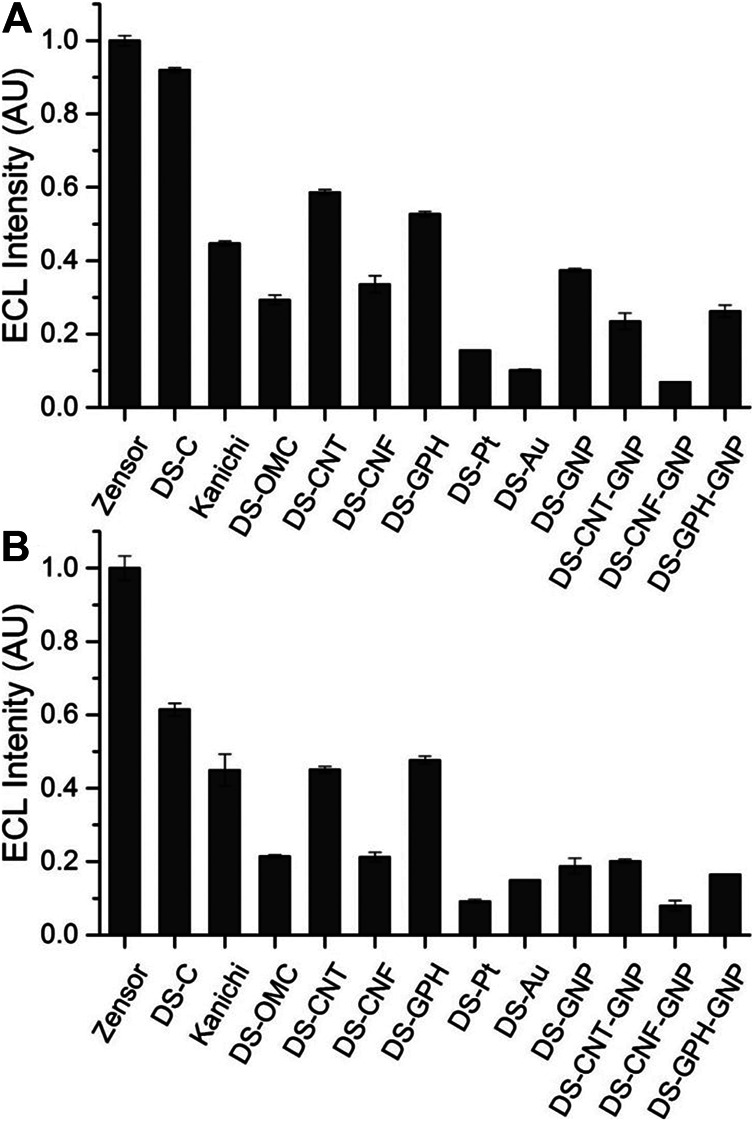
ECL intensities from various SPEs relative to Zensor, conducted in pH 7.5 PBS, 100 mM TPrA. **(A)** 1 × 10^−7^ M [Ru(bpy)_3_]^2+^. **(B)** 1 × 10^−8^ M [Ru(bpy)_3_]^2+^ (n = 3). Intensities are corrected for the difference in geometric working electrode area (Zensor and Kanichi SPEs 3 mm working electrode diameter, DS 4 mm working electrode diameter).

**FIGURE 4:**
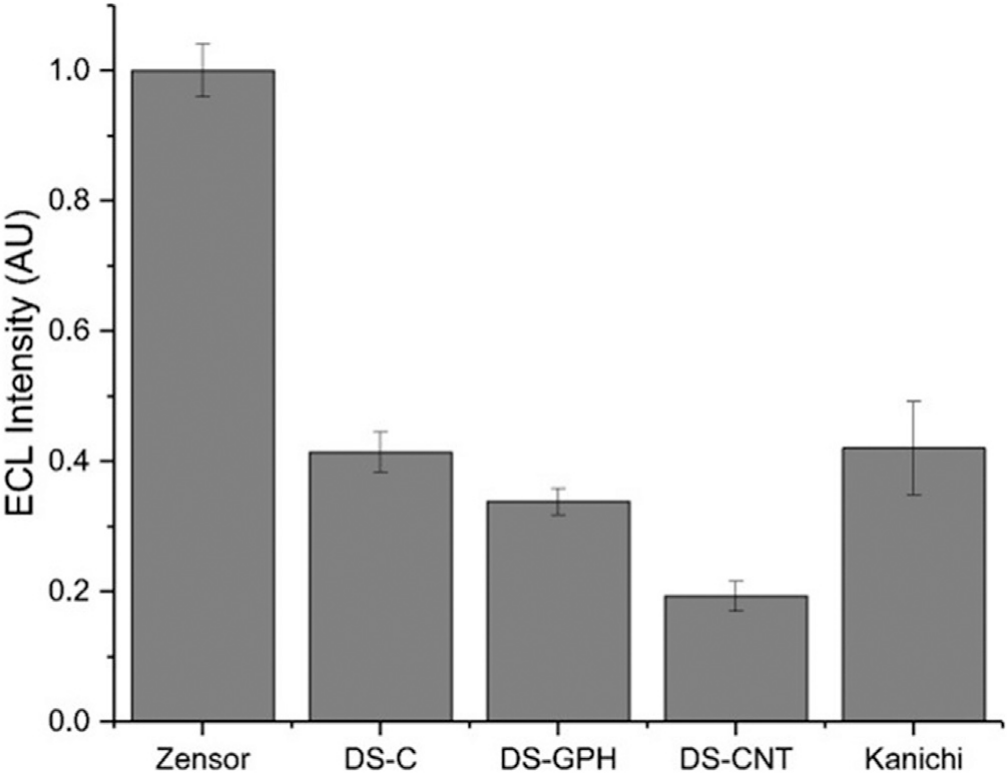
Relative ECL intensity (corrected for working electrode area) for bead-based DNA assay (100 mM TPrA, 0.2 M PBS, pH 7.5, n = 3).

We calculated ECL intensities relative to the Zensor carbon electrodes and corrected for differences in geometric working electrode area (using Eq. 1 in the SI; 3 mm diameter for Zensor and Kanichi SPEs; 4 mm diameter for DS varieties), as shown in Figure 3. DS-C and Zensor electrodes displayed the highest ECL intensities, followed in decreasing order by DS-CNT > DS-GPH > Kanichi > DS-GNP > DS-CNF > DS-OMC > DS-GPH-GNP > DS-CNT-GNP > DS-Pt > DS-Au and DS-CNF-GNP. This trend was consistent within experimental error at two concentrations (1 × 10^−7^ and 1 × 10^−8^ M). The five electrodes exhibiting the highest ECL intensities (Zensor, DS-C, DS-CNT, DS-GPH and Kanichi) were selected to carry out a magnetic bead-based DNA assay. We designed a bead-based assay using DNA bound to a magnetic bead to mimic a nucleic acid bead-based assay testing for a short length polymerase chain reaction (PCR) product. Using pre-bound DNA eliminates the experimental variability associated with primer recognition and binding; therefore, any variation in signal will result only from differences in the working electrode material, relative ECL intensities are summarized in Figure 4. Zensor electrodes displayed the highest relative ECL intensity followed by DS-C and Kanichi. A significant decrease in the relative ECL from both DS-CNT and DS-GPH electrodes in the bead-based assay experiments was observed, when compared to experiments with free complex.

Carbon-based electrodes are ideal for analytical ECL applications because they: have fast and efficient TPrA oxidation (Zu and Bard, 2000; Miao et al., 2002; Miao and Choi, 2004); are relatively hydrophobic (contact angle measurements for each electrode are included in Supplementary Table S1 (Workman and Richter, 2000; Li and Zu, 2004; Xu et al., 2005)), allowing high concentrations of TPrA to be present at the electrode surface; and have low rates of surface oxide formation compared to noble metal electrodes (Zu and Bard, 2000). These three factors result in the high ECL response we observed from carbon-based electrodes compared to DS-Pt, DS-Au, and electrodes with GNPs. Despite many publications employing nanostructured carbon materials for diagnostic ECL applications (Qi et al., 2009; Hu and Xu, 2010), including GNPs (Wang et al., 2006), OMC (Wu et al., 2014), GPH (Guo et al., 2016), and CNTs (Sardesai et al., 2011; Venkatanarayanan et al., 2012), we observed no enhancement of the ECL signal from electrodes composed of these materials in our experiments when compared to unmodified carbon SPEs, demonstrating that the incorporation of these nanomaterials is not necessarily advantageous for the [Ru(bpy)_3_]^2+^-TPrA ECL system.

Generally, nanomaterial electrodes exhibit superior ECL signals in assays where the functionality of the nanomaterial is integrated into the assay procedure (Zhao et al., 2002; Prieto et al., 2003; Carpini et al., 2004; Wang et al., 2006; Dong et al., 2007; Guo and Wang, 2007; Li et al., 2007; Liu et al., 2008; Forster et al., 2009; Qi et al., 2009; Sardesai et al., 2009; Hu and Xu, 2010; Malhotra et al., 2010; Xu et al., 2010; Sardesai et al., 2011; Zheng et al., 2011; Rusling, 2012; Venkatanarayanan et al., 2012; Liu and Song, 2014; Liu et al., 2016). For example, Guo et al. (2016) developed an ‘in-electrode’ biosensor by functionalizing two separate graphene sheets with a capture antibody (this sheet was then immobilized on the electrode surface) and a detection antibody (this secondary sheet was then functionalized with an electrochemiluminophore). When the bio-conjugate was assembled on the electrode surface, the two conductive graphene sheets served to extend the effective working electrode area, meaning all of the electrochemiluminophores were within the distance required for direct oxidation to produce ECL, unlike a conventional bead type assay, where the electrochemiluminophores may be located far outside of the electrode double layer or TPrA radical diffusion distance (Guo et al., 2016). Many nanomaterials also find applications with alternative co-reactants and luminophores (Dong et al., 2007; Guo and Wang, 2007; Qi et al., 2009; Rusling, 2012; Fang et al., 2018; Zhang et al., 2019; Zhang et al., 2020), which are beyond the scope of this evaluation. Although commercially available SPEs composed of carbon and nanomaterials showed no increase in ECL intensity when compared to unmodified carbon electrodes in our CV experiments; nanomaterials do present opportunities for innovative exploitation and modification when compared to classic carbon, platinum or gold electrodes; such as the aforementioned example. In the case of platinum and gold SPEs, and to a lesser extent in gold nanoparticle modified electrodes, a significant cathodic ECL signal was also observed, triggered by the reaction of [Ru(bpy)_3_]^2+^ with reactive oxygen species (ROS (Cao et al., 2002; Zheng et al., 2011; Lu et al., 2012; Li et al., 2013)). These ROS are formed upon reduction of the oxide layers at noble metal electrodes and the reduction of dissolved oxygen species. Negligible cathodic ECL was observed at carbon-based electrodes.

We selected CV, instead of chronoamperometry, for ECL generation because it provides information about the potential dependence of ECL processes; this is of particular interest to our research group (Doeven et al., 2013; Doeven et al., 2015b; Kerr et al., 2015; Kerr et al., 2016a; Kerr et al., 2016b; Soulsby et al., 2018a; Soulsby et al., 2018b). Furthermore, we observed higher variability in chronoamperometry experiments (average RSD 9%, n = 3) when compared to CV experiments (average RSD 3%, n = 3) and it was not possible to detect ECL using chronoamperometry from all electrodes at the same concentration (see Supplementary Figure S7). However, different relative ECL intensities were observed from chronoamperometry experiments (0.5 s pulse to 1.4 V vs Ag/AgCl) when compared to CV experiments; presumably, the reduced timespan of the applied potential minimizes electrode passivation and effects such as the electro-active surface area become more important for ECL intensity, indicating that the electrochemical technique/initiating voltage waveform can also have a significant effect on the ECL intensity generated from a specific electrode material.

### Effect of the Addition of Surfactants on ECL Intensity

Surfactants such as Tween 20 or Triton X-100 (Supplementary Figure S8) are regularly employed to enhance ECL at the noble metal electrode surfaces (Workman and Richter, 2000; Zu and Bard, 2000; Factor et al., 2001; Zu and Bard, 2001; Li and Zu, 2004; Xu et al., 2005). Surfactants increase the hydrophobicity of noble metal electrode surfaces, thereby increasing the concentration of TPrA at the electrode surface and facilitating higher rates of TPrA oxidation (Workman and Richter, 2000; Factor et al., 2001; Li and Zu, 2004; Xu et al., 2005). At carbon electrodes, researchers have demonstrated that surfactants have the opposite effect and the ready adsorption of the hydrophobic surfactant tails onto the electrode surface attenuates TPrA oxidation and ECL intensity (Xu et al., 2005).

**FIGURE 5:**
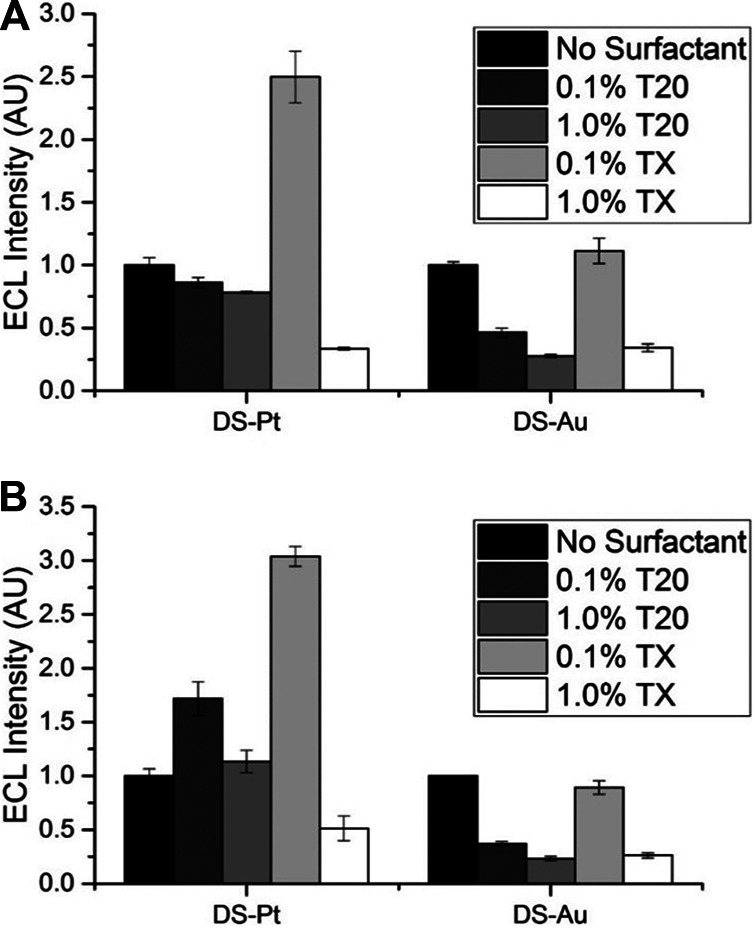
Effect of Tween 20 (T20) or Triton X-100 (TX) surfactants on ECL intensity relative to that from the same electrode without surfactant, 0.1 M PBS pH 7.5, 100 mM TPrA. **(A)** 1 × 10^−7^ M [Ru(bpy)_3_]^2+^. **(B)** 1 × 10^−8^ M [Ru(bpy)_3_]^2+^ (n = 3).

We investigated the effect of two surfactants, Tween 20 (T20) and Triton X-100 (TX), at concentrations of 0.1% and 1% at both DS-Pt and DS-Au electrodes on ECL intensity (Figure 5). Minimal enhancement at DS-Au electrodes was observed with the addition of surfactant, but DS-Pt electrodes exhibited up to 3-fold enhancement with the addition of 0.1% TX. Lower ECL intensities were observed at both DS-Pt and DS-Au electrodes with the addition of either 1% T20 or TX. As expected, no significant ECL enhancement was observed with the addition of surfactant at DS-C electrodes (Supplementary Figure S9; Workman and Richter, 2000; Zu and Bard, 2000; Factor et al., 2001; Li and Zu, 2004; Xu et al., 2005). Furthermore, with the addition of surfactant, the ECL intensity observed at carbon (DS-C) electrodes was ∼3-fold higher than that observed at DS-Pt electrodes and ∼8-fold higher than that observed at DS-Au electrodes with the addition of surfactant (0.1% TX).

### Stability of ECL Over Multiple Potential Cycles

Another important characteristic of electrodes for ECL applications is the stability of the ECL response over consecutive scans or potential cycles. Traditional solid disk electrodes are mechanically or electrochemically polished between ECL measurements to refresh the electrode surface and ensure reproducible results, but SPEs cannot be polished. To investigate this property, we conducted three potential scans at 0.1 V/s between 1.6 V to −1.2 V (vs Ag/AgCl) and measured the ECL response for each scan (Supplementary Figures S6, S10). Zensor, Kanichi and DropSens carbon electrodes generally displayed a consistent decrease in ECL with scan number. This results from a number of factors including deprotonation of the TPrA radical cation by oxygen containing surface species (Chen and Zu, 2008), increased oxidative consumption of the TPrA radical species (Chen and Zu, 2008), and passivation of the electrode surface caused by the attachment of dipropylamine (a side-product of TPrA^•^ oxidation) to the electrode surface after oxidation. A generalized reaction mechanism for this process has been included in the supplementary information (Adenier et al., 2004). The net result of these three factors is a steady passivation of the electrode surface, causing a decrease in ECL signal with scan number.

In contrast, we observed an increase in ECL response with scan number when using electrodes composed of platinum and certain carbon nanomaterials. In the case of platinum electrodes, this increase results from the intense cathodic ECL signal. In the case of SPEs modified with nanomaterials, it is possible that the nanomaterials may also be inherently stable and ‘resistant’ to passivation when compared to unmodified carbon (graphite) electrodes, or the nanomaterial may be subtly altered during the scanning process. When compared to a classic 3 mm GC electrode, we observed poor relative ECL intensities from commercially available SPEs; ranging from 4% for DS-Pt electrodes to 47% for Zensor electrodes (GC 100%). However, we observed a consistent decrease with scan number similar to that observed for unmodified carbon SPEs. Commercially available SPEs are considerably cheaper and easier to use (they do not require polishing), and therefore, SPEs are suitable for single-use experiments and applications where disposability is preferred; for example, dealing with bio-hazards (e.g., infectious viral agents), biological samples or in systems that are frequently contaminated (e.g., RNA assays).

## Conclusion

We have compared a variety of commercially available SPEs for their application in heterogenous and homogenous ECL sensing strategies. Unmodified carbon-based SPEs displayed the highest relative ECL intensities (Zensor 100%, DS-C 61%, and Kanichi 45%) when interrogated using CV. Surprisingly, for conventional bead-based ECL assay formats, these cheaper classic carbon SPEs provided the best performance. The incorporation of nanomaterials did not enhance the ECL signal when compared to unmodified carbonaceous SPEs. This study aims to aid researchers to choose the optimal SPE for their application from the plethora of commercially available varieties of SPEs. Our results suggest that unmodified carbon electrodes produce the brightest ECL using the conventional [Ru(bpy)_3_]^2+^ luminophore with TPrA co-reactant. Future studies elucidating the suitability of noble metal and nanomaterial modified electrodes for alternative sensing applications (e.g., incorporating electrografting or surface assembly of biological recognition elements) should be undertaken to determine the suitability of commercially available SPEs for a wider variety of sensing applications.

## Data Availability Statement

The original contributions presented in the study are included in the article/Supplementary Material, further inquiries can be directed to the corresponding authors.

## Author Contributions

EK conducted all experiments and data analysis and prepared the first draft of the manuscript. GB prepared labels for analysis. RA prepared electrode holders for analysis. EK, PF, and ED contributed to conceptualization. EK, RA, RG, PF, GB, and ED contributed to data interpretation and revision and editing of the manuscript. EK and PF obtained funding to undertake this work.

## Funding

The present work was carried out with the support of the Deakin Advanced Characterisation Facility. EK thanks National Health and Medical Research Council (NHMRC) of Australia (GNT1161573).

## Conflict of Interest

The authors declare that the research was conducted in the absence of any commercial or financial relationships that could be construed as a potential conflict of interest.
