# Supplementary material for: A Comparison of Commercially Available Screen-Printed Electrodes for Electrogenerated Chemiluminescence Applications
Source: Front Chem. 2021 Jan 28;8:628483. doi: 10.3389/fchem.2020.628483 (PMC7875866; doi:10.3389/fchem.2020.628483)
Supplement: Supplementary file 1 [file datasheet1.pdf]

Supplementary Material

1 Supplementary Information

Biotinylated 89mer ssDNA sequence: 5'- /5AmMC6/GAT GCA AGG TCG CAT ATG AGA TTT CTG TGG CAT CCT GGC GCT CCC CAC CAG TCT CCA TTT GTT CAT ATG ATC GTT TGG TGC CTT GAG AC/3Bio/ -3'.

General reaction mechanism for electrode fouling by dipropylamine (Adenier et al., 2004).

(1) $TPrA^{\bullet} - e^{-} \rightarrow Pr_2N^{+}C{=}H_2CH_3$

(2) $TPrA^{\bullet +} + TPrA^{\bullet} \rightarrow TPrA + Pr_2N^{+}C{=}H_2CH_3$

(3) $Pr_2N^{+}C{=}H_2CH_3 + H_2O \rightarrow Pr_2NH + CH_3CH_2CHO$

(4) $Pr_2NH - e^{-} \rightarrow Pr_2NH^{\bullet +}$

(5) $Pr_2NH^{\bullet +} \rightarrow Pr_2N^{\bullet} + H^{+}$

(6) 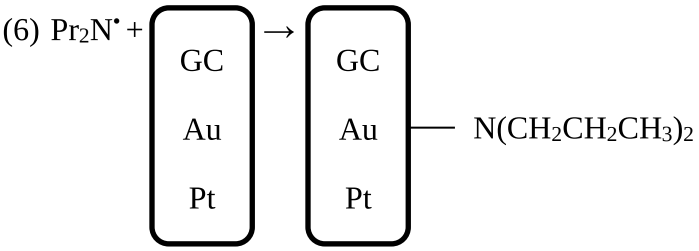

We used the following equation to account for differences in geometric working electrode area in order to calculate relative ECL intensities;

(1) $\left(\frac{ECL(SPE)}{A_{geo}(SPE)} \div \frac{ECL(Zensor)}{A_{geo}(Zensor)}\right) \times 100\%$

2 Supplementary Tables

Supplementary Table S1: Contact angle of water on different electrode surfaces.

Zensor	132° ± 1
DS-C	122° ± 1
Kanichi	132° ± 1
DS-OMC	125° ± 1
DS-CNT	107° ± 1
DS-CNF	150° ± 1
DS-GPH	117° ± 1
DS-Pt	99° ± 1
DS-Au	106.5 ± 1
DS-GNP	108° ± 1
DS-CNT-GNP	117° ± 1
DS-CNF-GNP	140° ± 1
DS-GPH-GNP	97° ± 1

3 Supplementary Figures

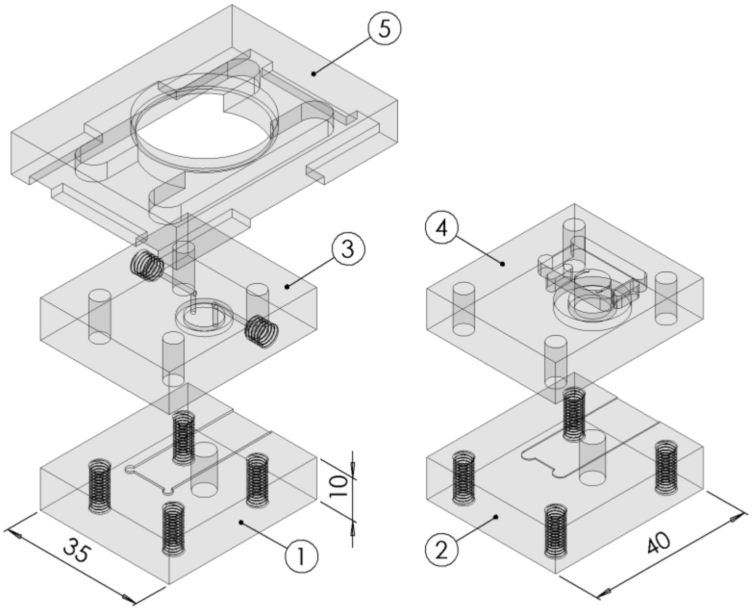

Supplementary Figure S1. CAD drawings of electrochemical cells used to house SPEs. (1,2) Base with hole below working electrode for magnet. (3) Flow cell for free-complex assays. (4) Cell with open top for bead-based assays. (5) Adaptor used to interface cells with PMT.

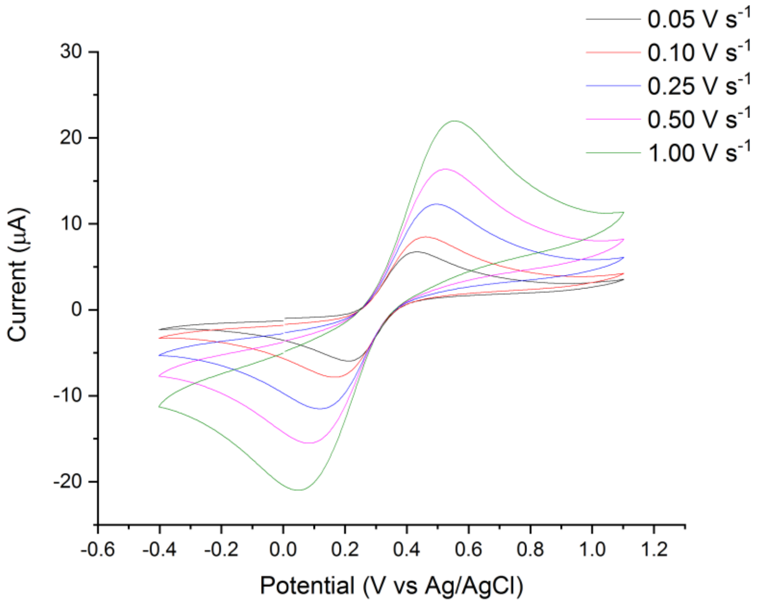

Supplementary Figure S2. Representative scan rate study of 1 mM potassium ferrocyanide in 1 M KCl at a Zensor electrode.

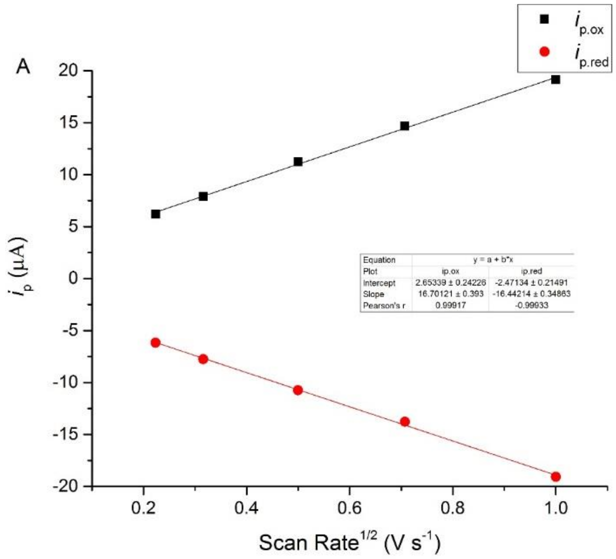

B

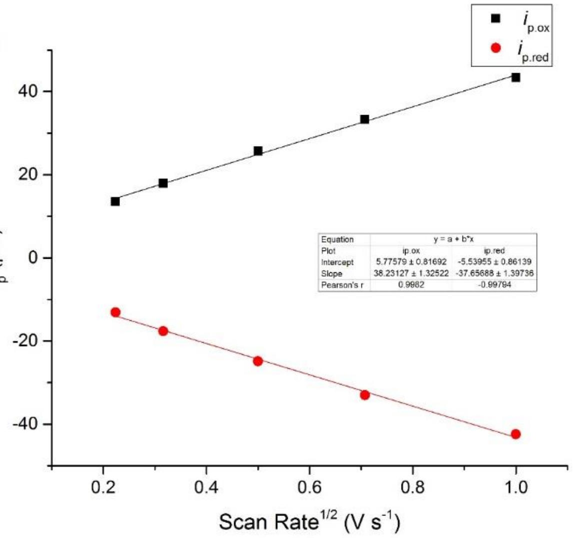
$i_{p.ox}$
$i_{p.red}$
Equation | y = a + b*x
Plot | ip.ox | ip.red
Intercept | 5.77579 ± 0.81692 | -5.53955 ± 0.86139
Slope | 38.23127 ± 1.32522 | -37.65688 ± 1.39736
Pearson's r | 0.9982 | -0.99794
i_p (μA)
Scan Rate$^{1/2}$ (V s^{-1})

C

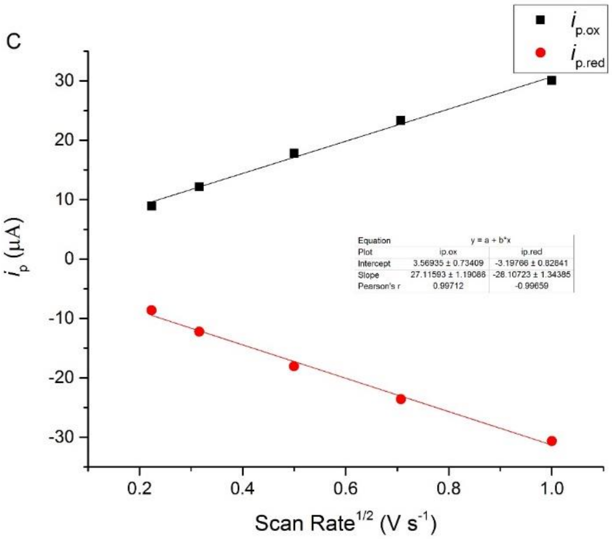
$i_{p.ox}$
$i_{p.red}$
Equation | y = a + b*x
Plot | ip.ox | ip.red
Intercept | 3.56935 ± 0.73409 | -3.19766 ± 0.82841
Slope | 27.11593 ± 1.19086 | -28.10723 ± 1.34385
Pearson's r | 0.99712 | -0.99659
i_p (μA)
Scan Rate$^{1/2}$ (V s^{-1})

D

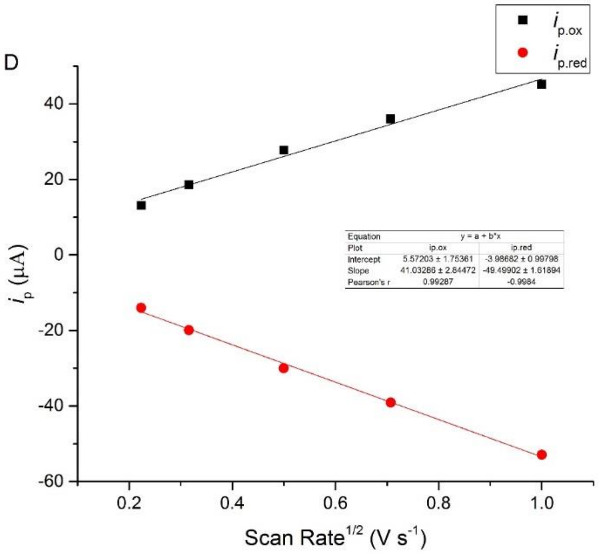
$i_{p.ox}$
$i_{p.red}$
Equation | y = a + b*x
Plot | ip.ox | ip.red
Intercept | 5.57203 ± 1.75361 | -3.98682 ± 0.99798
Slope | 41.03286 ± 2.84472 | -49.49902 ± 1.61894
Pearson's r | 0.99287 | -0.9984
i_p (μA)
Scan Rate$^{1/2}$ (V s^{-1})

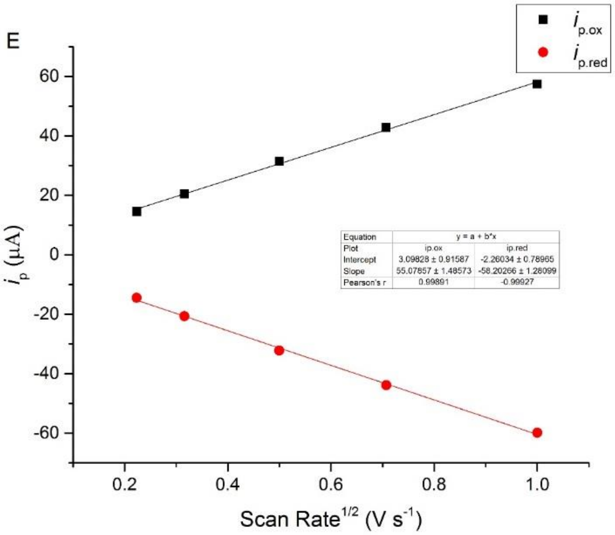
E
i p.ox
i p.red
Equation y = a + b*x
Plot ip.ox ip.red
Intercept 3.09828 ± 0.91587 -2.26034 ± 0.78965
Slope 55.07857 ± 1.48573 -58.20266 ± 1.28099
Pearson's r 0.99891 -0.99927
i p (μA)
Scan Rate1/2 (V s-1)

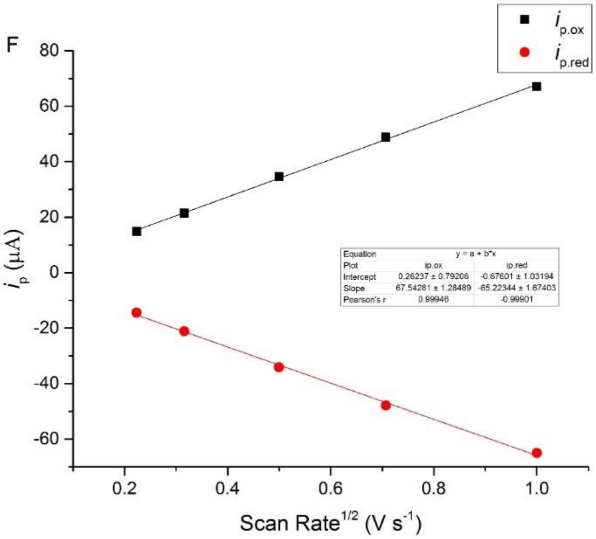
F
i p.ox
i p.red
Equation y = a + b*x
Plot ip.ox ip.red
Intercept 0.26237 ± 0.79206 -0.67601 ± 1.03194
Slope 67.54281 ± 1.28489 -65.22344 ± 1.67403
Pearson's r 0.99946 -0.99901
i p (μA)
Scan Rate1/2 (V s-1)

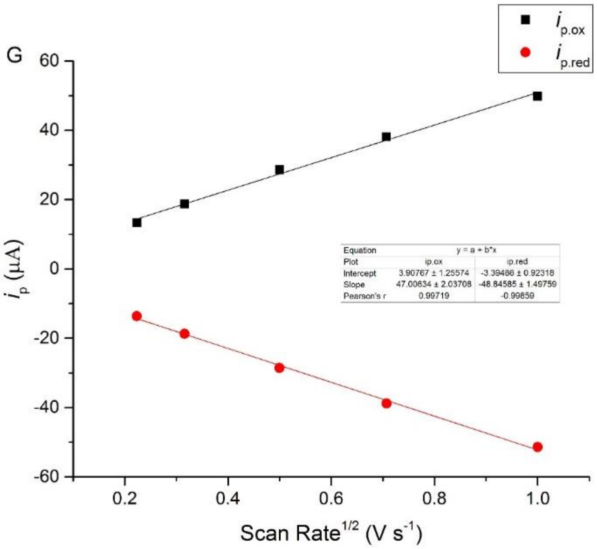
G
i p.ox
i p.red
Equation y = a + b*x
Plot ip.ox ip.red
Intercept 3.90767 ± 1.25574 -3.39486 ± 0.92318
Slope 47.00634 ± 2.03708 -48.84585 ± 1.49759
Pearson's r 0.99719 -0.99859
i p (μA)
Scan Rate1/2 (V s-1)

H

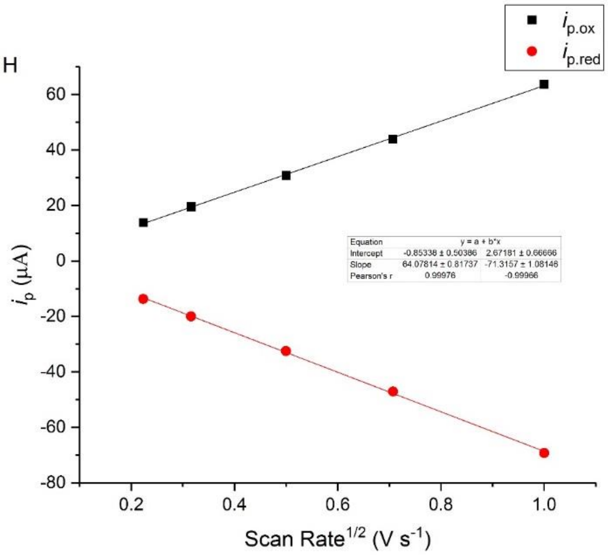

$i_{p.ox}$
$i_{p.red}$
i_p (μA)
Scan Rate$^{1/2}$ (V s^{-1})
Equation y = a + b*x
Intercept -0.85338 ± 0.50386 2.67181 ± 0.66666
Slope 64.07814 ± 0.81737 -71.3157 ± 1.08146
Pearson's r 0.99976 -0.99966

I

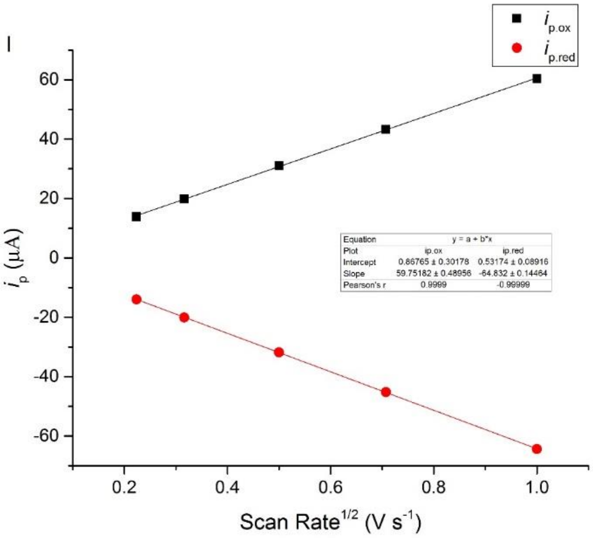

$i_{p.ox}$
$i_{p.red}$
i_p (μA)
Scan Rate$^{1/2}$ (V s^{-1})
Equation y = a + b*x
Plot ip.ox ip.red
Intercept 0.86765 ± 0.30178 0.53174 ± 0.08916
Slope 59.75182 ± 0.48956 -64.832 ± 0.14464
Pearson's r 0.9999 -0.99999

J

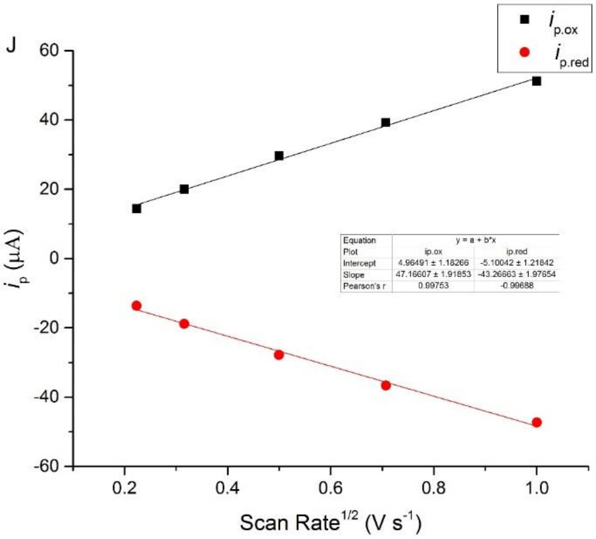

$i_{p.ox}$
$i_{p.red}$
i_p (μA)
Scan Rate$^{1/2}$ (V s^{-1})
Equation y = a + b*x
Plot ip.ox ip.red
Intercept 4.96491 ± 1.18266 -5.10042 ± 1.21842
Slope 47.16607 ± 1.91853 -43.26663 ± 1.97654
Pearson's r 0.99753 -0.99688

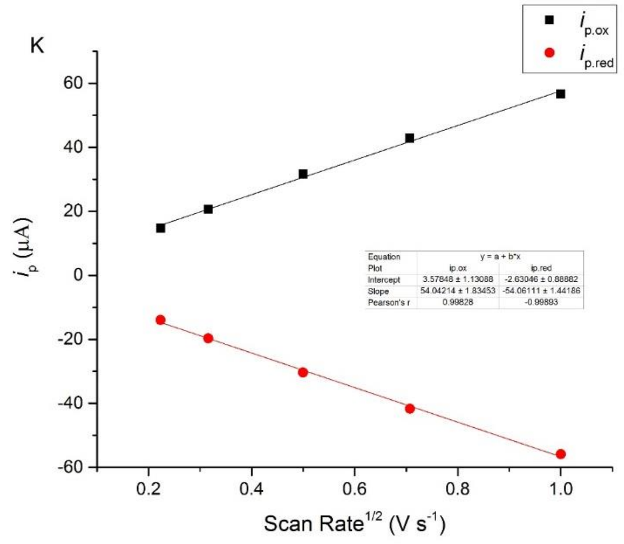
K
Equation | y = a + b*x
Plot | ip.ox | ip.red
Intercept | 3.57848 ± 1.13088 | -2.63046 ± 0.88882
Slope | 54.04214 ± 1.83453 | -54.06111 ± 1.44186
Pearson's r | 0.99828 | -0.99893
Scan Rate1/2 (V s-1)
ip (μA)

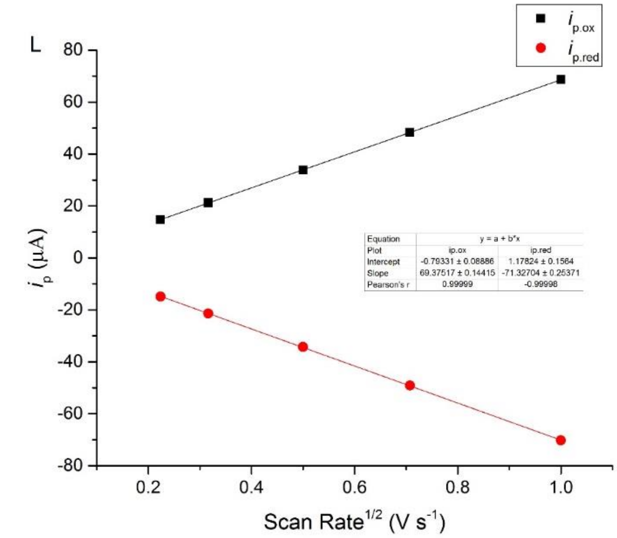
L
Equation | y = a + b*x
Plot | ip.ox | ip.red
Intercept | -0.79331 ± 0.08886 | 1.17824 ± 0.1564
Slope | 69.37517 ± 0.14415 | -71.32704 ± 0.25371
Pearson's r | 0.99999 | -0.99998
Scan Rate1/2 (V s-1)
ip (μA)

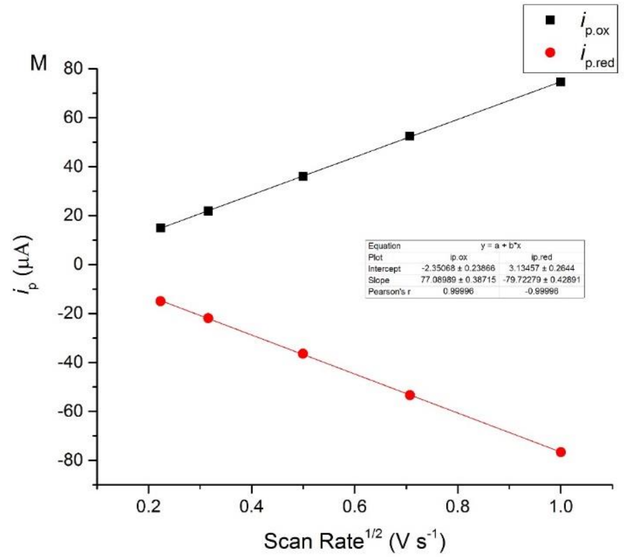
M
Equation | y = a + b*x
Plot | ip.ox | ip.red
Intercept | -2.35068 ± 0.23866 | 3.13457 ± 0.2644
Slope | 77.08989 ± 0.38715 | -79.72279 ± 0.42891
Pearson's r | 0.99996 | -0.99998
Scan Rate1/2 (V s-1)
ip (μA)

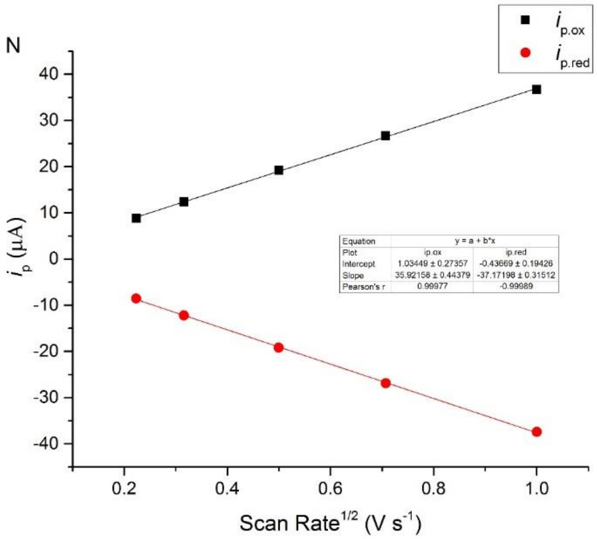

Supplementary Figure S3. Variation of peak current with scan rate, used to calculate the electroactive area of each electrode. Representative from 1 mM potassium ferrocyanide, 1 M KCl. (A) Zensor. (B) DS-C. (C) Kanichi. (D) DS-OMC. (E) DS-CNT. (F) DS-CNF. (G) DS-GPH. (H) DS-Pt. (I) DS-Au. (J) DS-GNP. (K) DS-CNT-GNP. (L) DS-CNF-GNP. (M) DS-GPH-GNP.

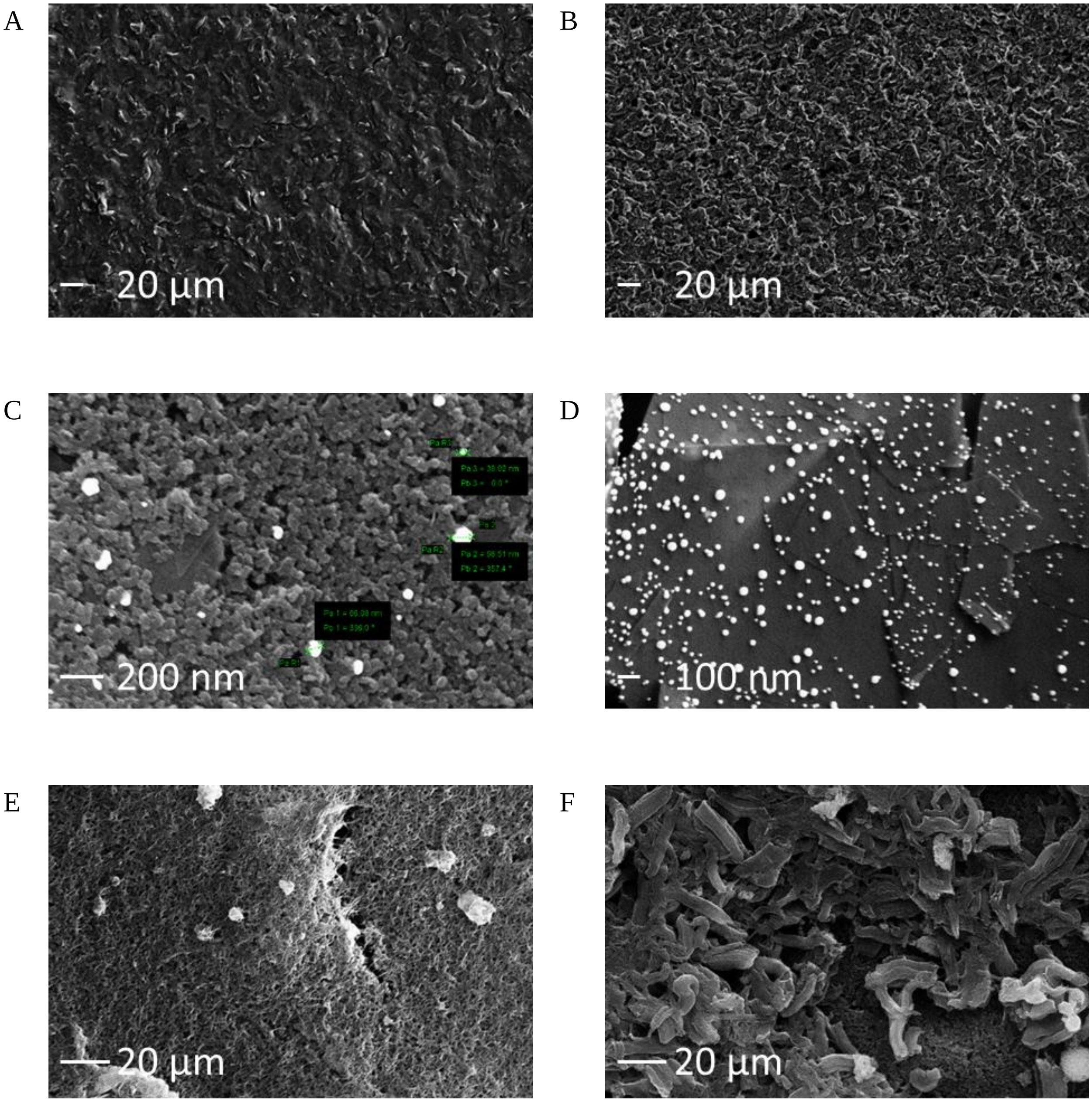

Supplementary Figure S4. SEM micrographs of commercially available SPEs. (A) DS-C. (B) Zensor. (C) DS-GNP, with measurements of GNP diameter. (D) DS-GPH-GNP. (E) DS-CNT. (F) DS-OMC. Figures A and B using secondary electron detector, all other micrographs obtained using in-lens detector.

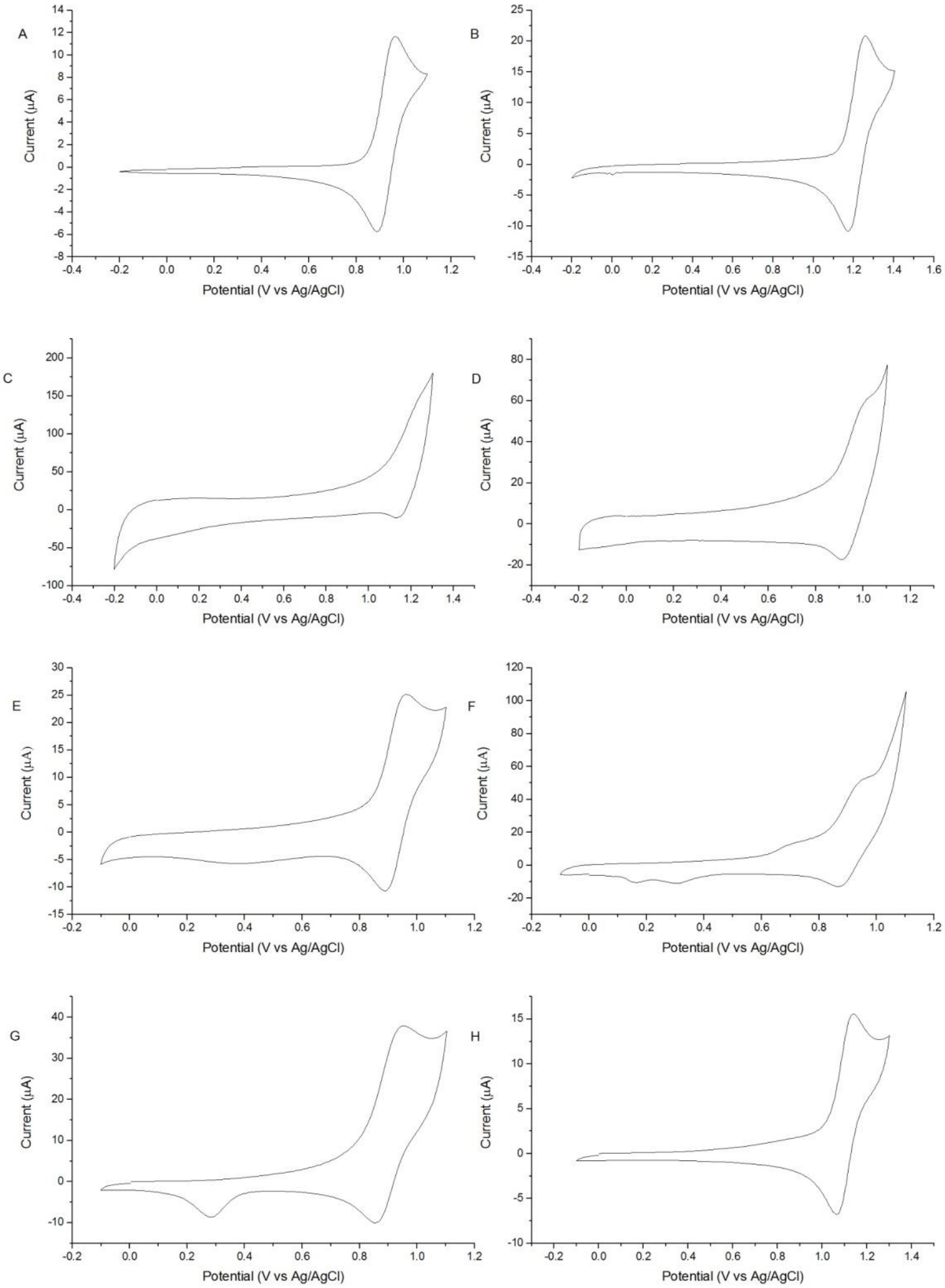

Supplementary Figure S5. CVs of 1 mM $[Ru(bpy)_3]^{2+}$ in 0.1 M PBS (scan rate 0.1 V/s). (A) Zensor. (B) DS-C. (C) DS-OMC. (D) DS-CNF. (E) DS-Pt. (F) DS-CNF-GNP. (G) DS-CNT-GNP. (H) GC.

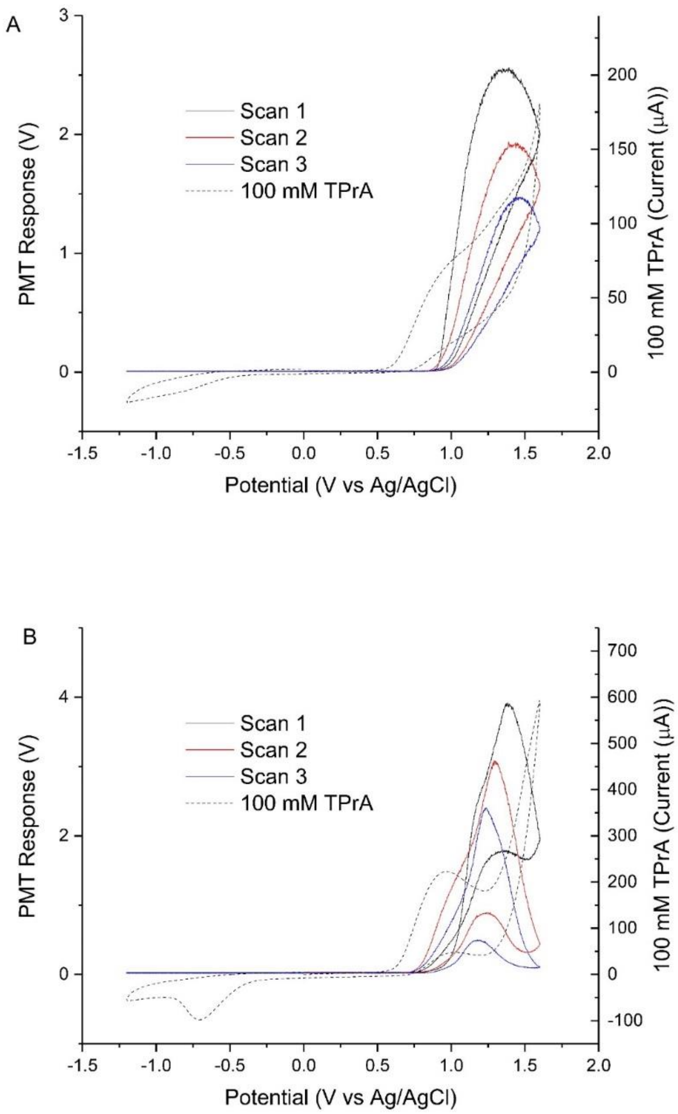

A
Scan 1
Scan 2
Scan 3
100 mM TPrA
PMT Response (V)
100 mM TPrA (Current (μA))
Potential (V vs Ag/AgCl)
B
Scan 1
Scan 2
Scan 3
100 mM TPrA
PMT Response (V)
100 mM TPrA (Current (μA))
Potential (V vs Ag/AgCl)

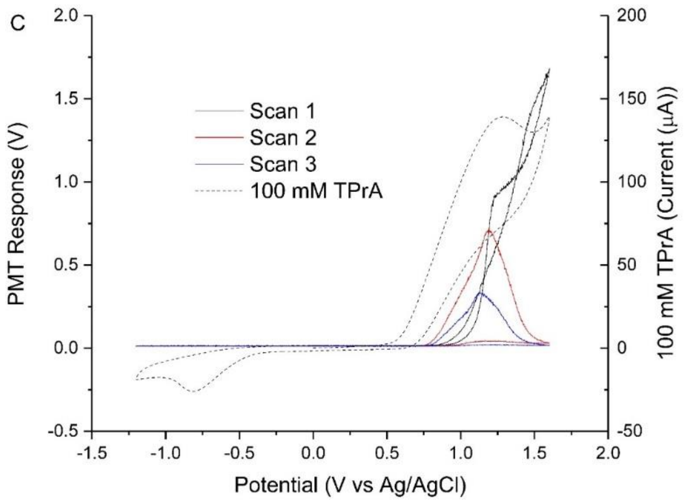
C
Scan 1
Scan 2
Scan 3
100 mM TPrA
PMT Response (V)
100 mM TPrA (Current (μA))
Potential (V vs Ag/AgCl)

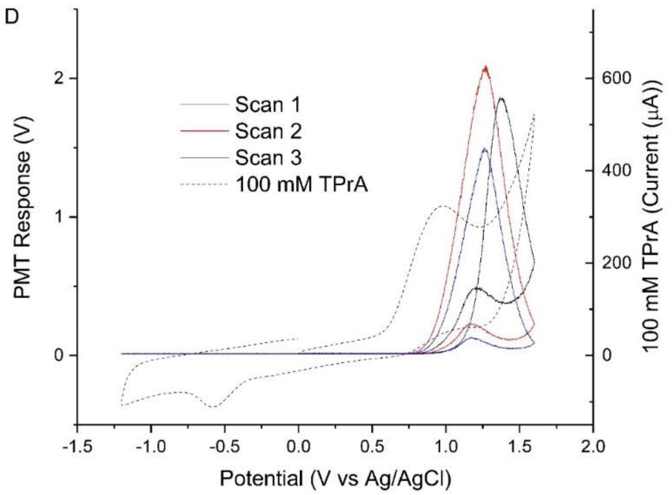
D
Scan 1
Scan 2
Scan 3
100 mM TPrA
PMT Response (V)
100 mM TPrA (Current (μA))
Potential (V vs Ag/AgCl)

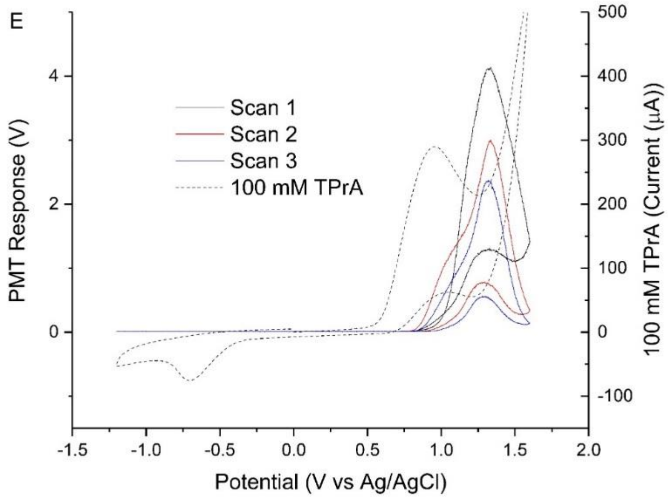
E
Scan 1
Scan 2
Scan 3
100 mM TPrA
PMT Response (V)
100 mM TPrA (Current (μA))
Potential (V vs Ag/AgCl)
-1.5
-1.0
-0.5
0.0
0.5
1.0
1.5
2.0
0
2
4
-100
0
100
200
300
400
500

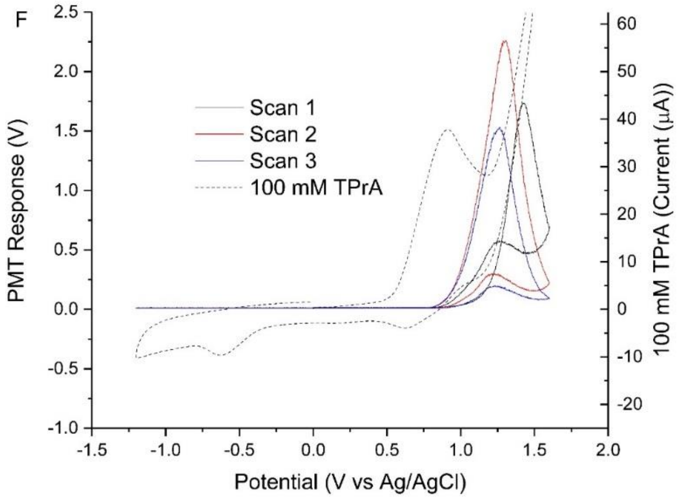
F
Scan 1
Scan 2
Scan 3
100 mM TPrA
PMT Response (V)
100 mM TPrA (Current (μA))
Potential (V vs Ag/AgCl)
-1.5
-1.0
-0.5
0.0
0.5
1.0
1.5
2.0
-1.0
-0.5
0.0
0.5
1.0
1.5
2.0
2.5
-20
-10
0
10
20
30
40
50
60

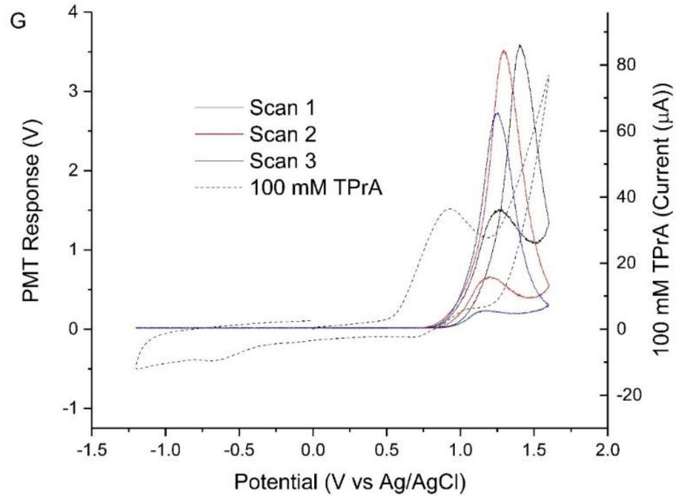
G
Scan 1
Scan 2
Scan 3
100 mM TPrA
PMT Response (V)
100 mM TPrA (Current (μA))
Potential (V vs Ag/AgCl)

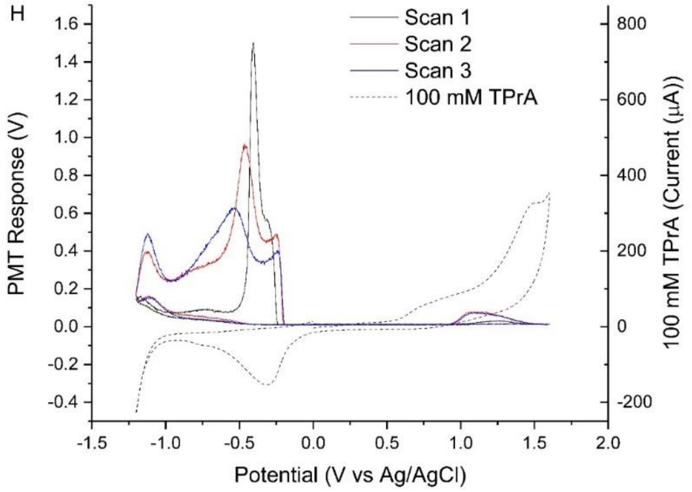
H
Scan 1
Scan 2
Scan 3
100 mM TPrA
PMT Response (V)
100 mM TPrA (Current (μA))
Potential (V vs Ag/AgCl)

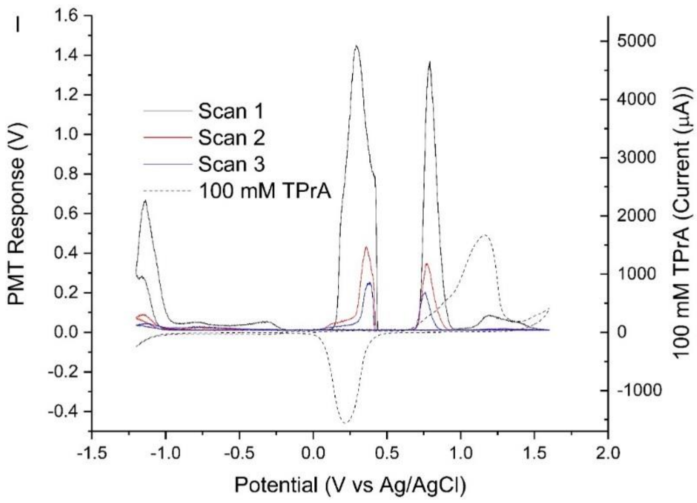
I
Scan 1
Scan 2
Scan 3
100 mM TPrA
PMT Response (V)
100 mM TPrA (Current (μA))
Potential (V vs Ag/AgCl)

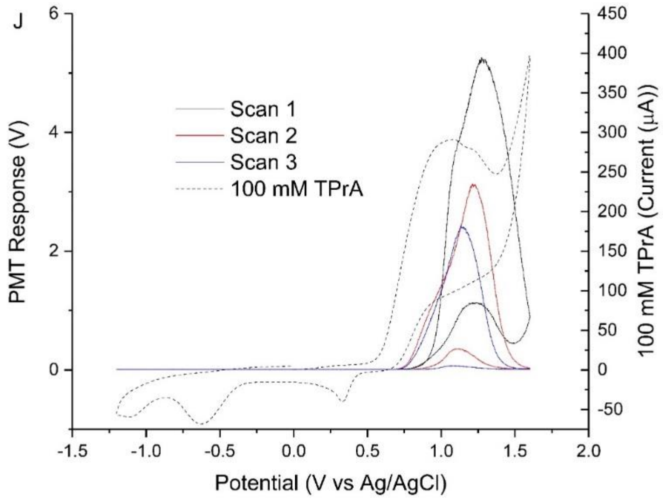
J
Scan 1
Scan 2
Scan 3
100 mM TPrA
PMT Response (V)
100 mM TPrA (Current (μA))
Potential (V vs Ag/AgCl)

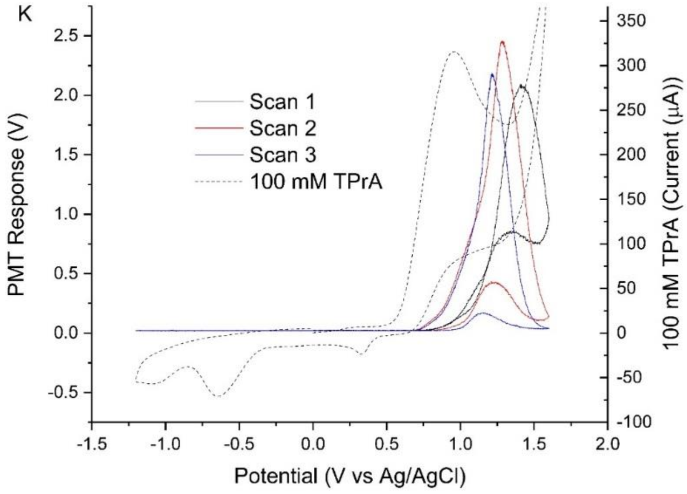
K
Scan 1
Scan 2
Scan 3
100 mM TPrA
PMT Response (V)
100 mM TPrA (Current (μA))
Potential (V vs Ag/AgCl)

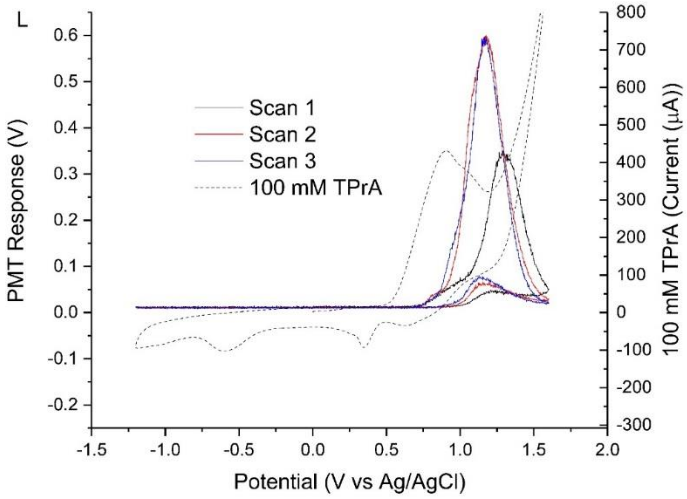
L
Scan 1
Scan 2
Scan 3
100 mM TPrA
PMT Response (V)
100 mM TPrA (Current (μA))
Potential (V vs Ag/AgCl)

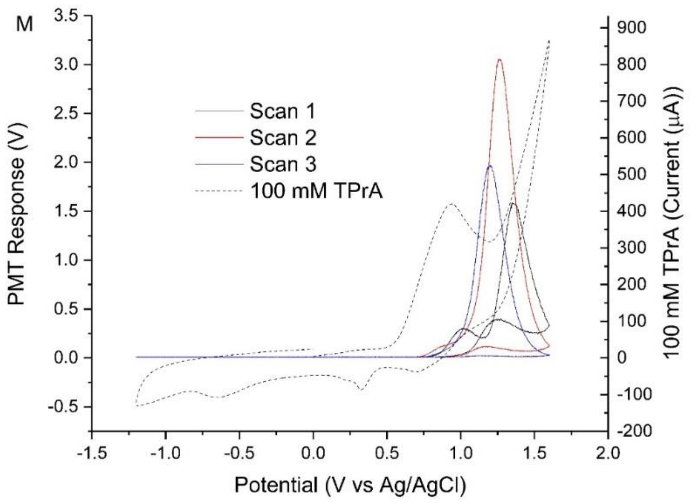

Supplementary Figure S6. Representative ECL response from 1 × 10^{-7} M $[Ru(bpy)_3]^{2+}$, 100 mM TPrA, 0.1 M PBS, pH 7.5 at different electrodes (solid lines, primary axis) and a representative CV of 100 mM TPrA in 0.1 M PBS (dotted line, secondary axis). (A) Zensor. (B) DS-C. (C) Kanichi. (D) DS-OMC. (E) DS-CNT. (F) DS-CNF. (G) DS-GPH. (H) DS-Pt. (I) DS-Au. (J) DS-GNP. (K) DS-CNT-GNP. (L) DS-CNF-GNP. (M) DS-GPH-GNP.

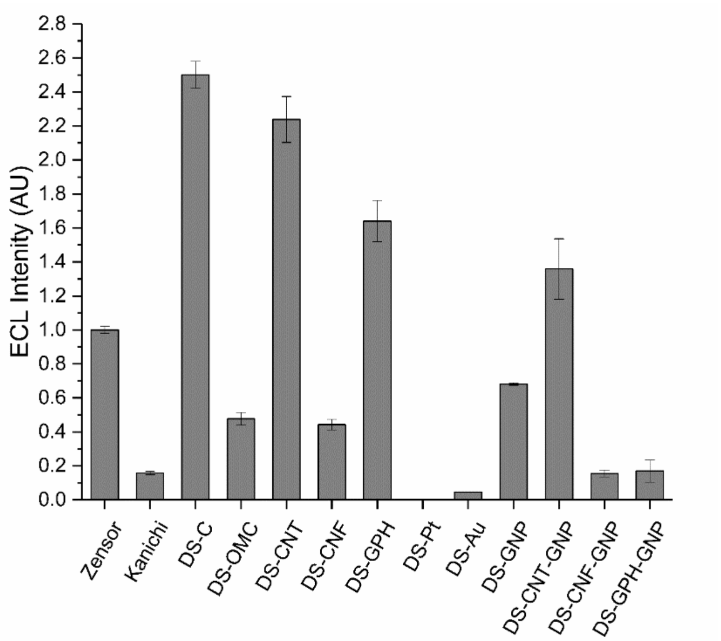

Supplementary Figure S7. ECL response from chronoamperometry experiments. The signal is the integrated area from a 0.5 s pulse to 1.4 V vs Ag/AgCl. Each with 100 mM TPrA, 0.1 M PBS, pH 7.5, 2 × 10^{-7} M $[Ru(bpy)_3]^{2+}$. No ECL response from DS-Pt electrodes was observed at 2 × 10^{-7} M. The ECL response from Kanichi was insignificant compared to the background response at 2 × 10^{-7} M $[Ru(bpy)_3]^{2+}$; therefore, we compared Kanichi electrodes relative to the response of Zensor at 1 × 10^{-6} M. All ECL responses are corrected for differences in geometric working electrode area.

A

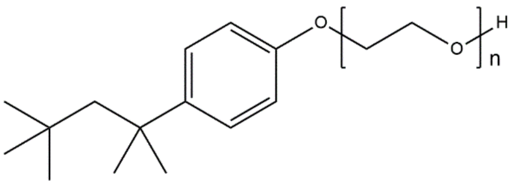

B

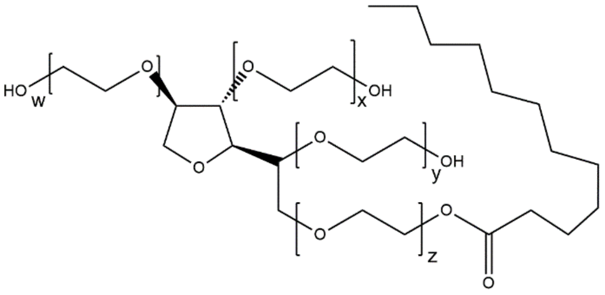

Supplementary Figure S8. (A) Triton X-100 (TX; n = 9-10) and (B) Tween 20 (T20; w + x + y + z = ~20).

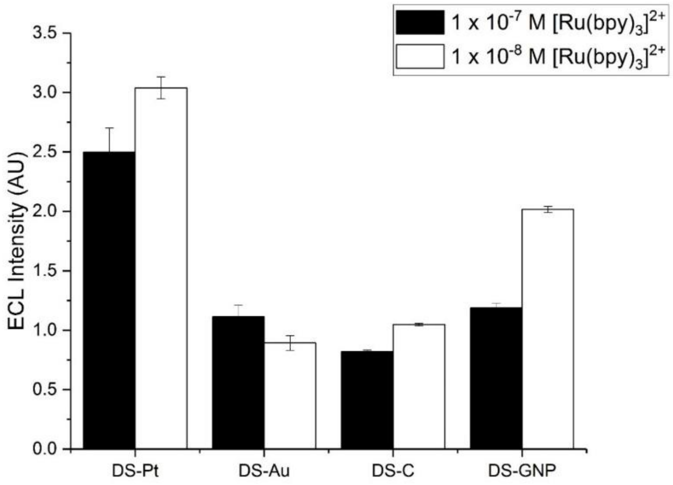

Supplementary Figure S9. Effect of 0.1% TX on ECL intensity, relative to ECL response from each electrode variety with no surfactant (0.1 M PBS, pH 7.5, 100 mM TPrA, n = 3).

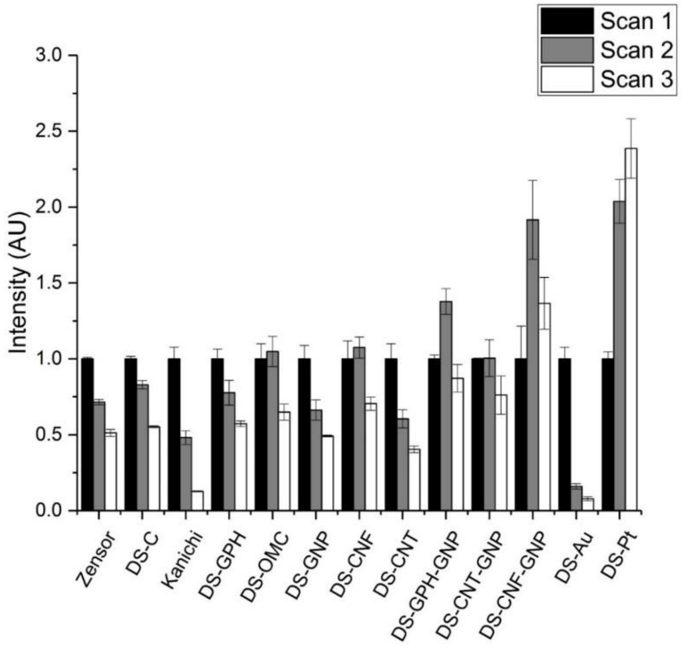

Supplementary Figure S10. Variation of ECL response with scan number. Scans 2 and 3 are calculated relative to scan 1 for each electrode variety. Each with 100 mM TPrA, 0.1 M PBS, pH 7.5, 1×10^{-7} M $[Ru(bpy)_3]^{2+}$.

References

Adenier, A., Chehimi, M.M., Gallardo, I., Pinson, J., and Vila, N. (2004). Electrochemical oxidation of aliphatic amines and their attachment to carbon and metal surfaces. *Langmuir* 20(19)**,** 8243-8253. doi: 10.1021/la049194c.
